# The interrelations between psychological outcome trajectories and resource changes amid large-scale disasters: A growth mixture modeling analysis

**DOI:** 10.1038/s41398-023-02350-4

**Published:** 2023-02-15

**Authors:** Tiffany Junchen Tao, Li Liang, Huinan Liu, Stevan E. Hobfoll, Wai Kai Hou, George A. Bonanno

**Affiliations:** 1 Centre for Psychosocial Health, The Education University of Hong Kong, Hong Kong SAR, China; 2 Department of Psychology, The University of Hong Kong, Hong Kong SAR, China; 3 Department of Psychology, The Education University of Hong Kong, Hong Kong SAR, China; 4 STAR Consultants-STress, Anxiety and Resilience, Salt Lake City, UT USA; 5 Department of Counseling and Clinical Psychology, Teachers College, Columbia University, New York, NY 10027 USA

**Keywords:** Psychiatric disorders, Human behaviour, Scientific community

## Abstract

Currently little is known about the interrelations between changes in psychiatric symptoms and changes in resources (personal, social, financial) amid large-scale disasters. This study investigated trajectories of psychiatric symptoms and their relationships with different patterns of changes in personal, social, and financial resources between 2020 and 2022 amid the COVID-19 pandemic. A population-representative sample (*N* = 1333) was recruited to complete self-report instruments at the pandemic’s acute phase (February–July 2020, T1), and again at 1-year (March–August 2021, T2) and 1.5-year (September 2021–February 2022, T3) follow-ups. Respondents reported depressive and anxiety symptoms, self-efficacy, perceived social support, and financial capacity. Growth mixture modeling (GMM) identified four trajectories of depressive and anxiety symptoms: *resilience* (72.39–74.19%), *recovery* (8.40–11.93%), *delayed distress* (7.20–7.35%), and *chronic distress* (8.33–10.20%). Four patterns were demonstrated in resource changes: persistent high resources (40.89–47.64%), resource gain (12.08–15.60%), resource loss (6.30–10.43%), and persistent low resources (28.73–36.61%). Loss and gain in financial resources characterized *chronic distress* and *resilience*, respectively. Loss in personal resources characterized *delayed distress*, whereas loss or no gain in social resources was related to *chronic/delayed distress*. Respondents in *resilience* were also more likely to have persistent high resources while those with *delayed/chronic distress* were more likely to have persistent low resources. These results provide an initial evidence base for advancing current understanding on trajectories of resilience and psychopathology in the context of resource changes during and after large-scale disasters.

## Introduction

Under exposure to highly disruptive and potentially life-threatening events, individuals display heterogeneity in psychological outcomes, ranging from *resilience*, denoting stable patterns of low psychopathology or high well-being, to *chronic distress*, denoting persistent clinically significant psychiatric symptoms [1, 2]. Over the past two decades, four trajectories have been consistently identified across different contexts, such as military experiences, war and displacement, civilian accidents, bereavement, and major illnesses [3]. The majority of individuals displayed *resilience* (35–65%), whereas others displayed *recovery*, denoting improvement of symptoms from clinically significant to subclinical levels (15–25%), *delayed distress*, denoting deterioration of symptoms from initial normative to clinical levels (0–15%), and *chronic distress*, denoting persistent high scores above the clinical cut-offs (5–30%). There is growing evidence suggesting that psychological resilience is also common in adaptation to COVID-19 [4]. Between April and October, 2020, in Wuhan China following the COVID-19 outbreak, the majority of respondents (58–93%) demonstrated psychological resilience, i.e., clinically non-significant depressive, anxiety, and PTSD symptoms across four waves of assessment [5]. Similarly, resilience in depressive and anxiety symptoms was demonstrated in 60% Polish respondents between May 2020 and April 2021 [6] and 62–73% in Israeli respondents between May and October 2020 [7].

What is less known is the prospective interrelations between trajectories of psychopathology and resilience and resource changes under the COVID-19 context. A consensus among resilience-related frameworks is that individuals’ possession of and access to resources create a context for positive psychological adjustment [8–10]. According to the Conservation of Resources (COR) theory [11], resources are embedded within personal characteristics, energies, interpersonal relationships, and interactions, objects, and conditions, whereas stress adaptation is fundamentally driven by the pursuit of resource gain and avoidance of resource loss. From such conceptual standpoint, resilience is foremost a property of the environment where its individuals both have access to rich resources and are protected from resource loss [12, 13].

Theoretical and empirical evidence has established the relatively more salient weight of resource loss (vs. gain) in predicting adaptation outcomes [12, 14, 15]. Additionally, resources do not exist independently but establish or dissolve in aggregation. Resource gain (loss) begets further resource gain (loss) in the same direction, forming gain (loss) spirals. These spirals, denoting the nurturance or the blockage of resource accumulation, impact mental health through consolidating or eroding other resources [14, 15]. Therefore, resilience could be conceived as the optimal product of an interconnected system of numerous factors residing upon different layers of the socioecological structure, and these factors further interact with and activate one another to form an intricately dynamic mechanism [16, 17]. Accompanying the surge in research interest in resilience and resource changes, research on stress and resilience has demonstrated a shift of focus from internal attributes to contexts of individual strengths, an important advancement that acknowledges and considers the essential complexity of resilience [13, 17, 18]. Characteristics of the social ecology could get under the skin of individuals, activating individual attributes that in turn relate to resilient outcomes [12, 13, 19].

Resource changes have been closely related to individuals’ adjustment across different large-scale disasters. Over the six years following a large-scale earthquake in Japan, social and city-level support were found to predict post-disaster recovery from psychological distress [20]. In the aftermath of the 9-11 terrorist attack, high social support and income (as well as no income decline) related to the absence of probable PTSD [8]. Following the mass-scale terrorism in Israel [21], perceived social support positively predicted whereas psychosocial and financial resource losses negatively predicted resilience and recovery (relative to chronic distress) on depressive and PTSD symptoms.

In-depth investigation is nonetheless needed on changes in resources as a dynamic factor in determining longitudinal trajectories of psychopathology and resilience within the current COVID-19 pandemic [11, 22–24]. Cross-sectional evidence is available to show that COVID-19-induced resource losses in psychological, interpersonal, financial, and self-care aspects were positively associated with higher depressive, anxiety, and peritraumatic symptoms, or general distress among discharged COVID-19 inpatients in China [25], home-bound older adults in the US [26], people with chronic diseases in the US [26], and Syrian refugees [27]. A 10-week prospective study showed that perceived social support predicted subsequent higher well-being, lower distress, and decreased distress over the period among an online American sample (*n* = 674) [28]. Prospective studies have also reported that lower socioeconomic resources at the outset of the pandemic, be it low income or high financial hardship, predicted persistent high distress or increased distress in the years following the outbreak [6, 7, 29].

This study aimed to investigate the heterogeneity of trajectories of depressive and anxiety symptoms among a population-representative cohort over 1.5 years following the COVID-19 outbreak, and how different changes in personal, social, and financial resources distinctively characterized these outcome trajectories. Based on previous evidence on outcome trajectories and the COR theory, the following hypotheses were tested:

### *Hypothesis 1*.

The four prototypical trajectories of clinical symptoms, *resilience*, *recovery*, *delayed distress*, and *chronic distress*, will emerge among the individuals in the 1.5 years following the outbreak of COVID-19.

### *Hypothesis 2*.

Individuals who have higher levels of resources or gain in resources over time will be more likely to follow the *resilience* and *recovery* trajectories than the *delayed distress* or *chronic distress* trajectories.

### *Hypothesis 3*.

Individuals who have lower levels of resources or loss in resources will be more likely to follow the *delayed distress* or *chronic distress* trajectories than the *resilience* and *recovery* trajectories.

## Materials and methods

### Respondents and procedures

This was a prospective cohort study with data collected at three time points, February–July 2020 (T1), March–August 2021 (T2), and September 2021–February 2022 (T3). Upon obtaining the approvals from the Ethics Review Committee of The Education University of Hong Kong, telephone surveys were conducted to recruit and assess population-representative Hong Kong Chinese aged ≥15 years at T1. Verbal informed consent was obtained from each participant prior to the survey. The sampling procedure at T2 followed closely that of other large-scale local prospective cohort study [30–32]. Respondents at T1 were randomly invited to participate in the 1-year (T2) and 1.5-year (T3) follow-ups. The final sample included 1333 respondents (T1: *N* = 1333; T2: *N* = 1318; T3: *N* = 906). To handle missing data, we adopted a combination of missing imputation and full information maximum likelihood estimations in subsequent analyses to fully utilize available information. The cooperation (i.e., eligible individuals invited) and response (i.e., invited individuals complying with acceptable standards) rates were 91.72% and 76.54% at T2, and 98.48% and 68.64% at T3. Differences in sociodemographic characteristics between invited and non-invited respondents were non-significant. Detailed sampling information is documented in Supplementary Material 1. All respondents received supermarket coupons with face value HK$100 (≈US$13) as compensation for each participation.

### Measures

#### Demographics

Respondents reported demographic information (i.e., age, gender, education level, marital status, employment status, and monthly household income) with a standardized proforma at T1.

#### Personal resources

Self-efficacy was measured using the Chinese version of the 6-item General Self-Efficacy Scale (GSE-6) [33] at T2 and T3. Respondents rated their perceived ability to take control under stressful conditions *over the past month* on a 4-point Likert scale (1 = strongly disagree, 4 = strongly agree). Higher total scores indicated higher levels of self-efficacy (range = 6–24). Personal resources were defined as traits and/or skills that aid stress resistance [34]. Among them, self-efficacy, denoting one’s perceived capacity to cope with stressors [35], has been consistently found to buffer individuals against the adverse impact of disasters [36]. As a short form of the original 10-item version, the GSE-6 has displayed good reliability and validity across Chinese populations [37, 38]. Cronbach’s αs in the current administration were 0.879 (T2) and 0.859 (T3).

#### Social resources

Perceived social support was measured using the 12-item Multidimensional Scale of Perceived Social Support (MSPSS) [39] at T2 and T3. Respondents rated their perceived social support from family, friends, and a significant other *over the past month* on a 6-point Likert scale (1 = strongly disagree, 6 = strongly agree). Higher summed scores indicated greater perceived social support (range = 12–72). The MSPSS demonstrated good psychometric properties among the Chinese population [40]. Cronbach’s αs in the current administration were 0.940 (T2) and 0.928 (T3).

#### Financial resources

Financial capacity was measured at T2 and T3 with the 9-item Perceived Economic Strain Scale from the Economic Strain Model [41]. Respondents were asked to report on difficulties they had in affording living necessities or optional accoutrements (e.g., food, clothing, furniture, leisure activities) *over the past six months* in eight items on a 4-point scale (1 = strongly disagree, 4 = strongly agree). Respondents also reported the amount of money left at the end of the month *over the past six months* on a 4-point scale (1 = more than enough money left over, 4 = not enough to make ends meet). Scores were reverse coded with higher scores indicating higher financial capacity (range = 9–36). Similar items have displayed good psychometric properties among Chinese populations [42, 43]. The αs in the current administration were 0.886 (T2) and 0.877 (T3).

#### Depressive symptoms

Depressive symptoms were assessed using the 9-item Patient Health Questionnaire (PHQ-9) [44] across the three timepoints. Respondents indicated the frequency of experiencing depression-related symptoms *over the past two weeks* on a 4-point scale (0 = not at all, 1 = on several days, 2 = on more than half of the days, 3 = nearly every day). Higher total scores indicated greater severity of depressive symptoms (range = 0–27). Probable depression was indicated by a score of 10 or above [45]. The PHQ-9 has demonstrated good reliability and validity among Chinese populations [46, 47]. It also demonstrated high internal consistency at T1 (α = 0.832), T2 (α = 0.890), and T3 (α = 0.880) in the current study.

#### Anxiety symptoms

Anxiety symptoms were assessed using the 7-item Generalized Anxiety Disorder scale (GAD-7) [48] across the three timepoints. Respondents indicated the frequency they experienced anxiety symptoms *over the past two weeks* on a 4-point scale (0 = not at all, 1 = on several days, 2 = on more than half of the days, 3 = nearly every day). Higher summed scores indicated greater severity of anxiety symptoms (range = 0–21). Probable anxiety was indicated by a score of 10 or above [49]. The GAD-7 showed good psychometric properties in studies of Chinese populations [50–52] and showed high internal consistency at T1 (α = 0.923), T2 (α = 0.941) and T3 (α = 0.940) administrations.

### Statistical analysis

Missing data were handled with multiple imputation in SPSS Version 25. All main analyses were performed in M*plus* 8.3 [53] with full information maximum likelihood (FIML) estimation. To identify latent trajectories of depressive and anxiety symptoms over the three timepoints T1 through T3, a series of unconditional growth mixture models (GMM) were created. To facilitate model convergence, we allowed the variance of the intercept to be freely estimated while fixing the slope parameter. After selecting the optimal unconditional model, the Bolck, Croon, and Hagenaars (BCH) method [54, 55] was used to obtain the conditional models for the depressive and anxiety trajectories. The conditional models tested the following predictors: age, gender, education level, marital status, employment status, monthly household income, and change patterns in resources (personal, social, financial). To identify patterns of resource changes from T2 to T3, scores on personal, social, and financial resources were recoded into high or low based on median split at each timepoint (with reference to a previous study [56]), of which different combinations generated four categories: low-low (persistent low resources), low-high (resource gain), high-low (resource loss), and high-high (persistent high resources). The associations between symptom trajectories and predictors were estimated by regressing latent class variables on the predictors with multinomial logistic regression.

The growth mixture modeling analysis was conducted using the maximum likelihood estimator with robust standard error (MLR). Model solutions were evaluated based on the Bayesian Information Criteria (BIC) and sample-size-adjusted Bayesian Information Criteria (SABIC). Lower values of these indices indicate better fit. The Lo-Mendell-Rubin adjusted likelihood ratio test (LMR-LRT) and bootstrapped likelihood ratio test (BLRT) evaluated whether an additional class would improve model fit. A non-significant *p*-value of model with *k* classes supported the selection of model with *k*-1 class(es). Higher values of entropy demonstrated better classification quality. Fit statistics might not congruently point to a single solution, therefore fit statistics, interpretability, and theoretical relevance were jointly considered to determine the final decisions [57].

## Results

### Respondents and descriptive characteristics

Descriptive statistics of the respondents (*N* = 1333) are summarized in Table 1. The sample-level mean scores of self-efficacy, perceived social support, and financial capacity were 15.82 (*SD* = 3.31), 52.64 (*SD* = 11.70), and 24.63 (*SD* = 5.21) at T2 and 15.85 (*SD* = 2.73), 51.47 (*SD* = 9.49), and 24.35 (*SD* = 4.56) at T3. On a sample level, respondents experienced a decrease in perceived social support (*p* < 0.001) and financial capacity (*p* = 0.006) but not self-efficacy (*p* = 0.698). Considering individual differences in change patterns, about half of respondents had persistent high self-efficacy, perceived social support, and/or financial capacity (40.89–47.64%), 28.73–36.61% with persistent low resources, 12.08–15.60% with resource gain, and 6.30–10.43% with resource loss.

**Table 1:** Descriptive characteristics of the current sample.

Variable	*n* (%)	*Mean* (*SD*)	*p*
**Gender**			
Male	684 (51.31%)	–	–
Female	649 (48.69%)	–	–
**Age**			
55 or above	341 (25.58%)	–	–
45–54	183 (13.73%)	–	–
35–44	241 (18.08%)	–	–
25–34	306 (22.96%)	–	–
15–24	262 (19.65%)	–	
**Education**			
Tertiary or above	784 (58.81%)	–	–
Secondary or below	549 (41.19%)	–	–
**Marital status**			
Married	647 (48.54%)	–	–
Single/divorced/widowed	686 (51.46%)	–	–
**Employment**			
Employed	791 (59.34%)	–	–
Dependent	478 (35.86%)	–	–
Unemployed	64 (4.80%)	–	–
**Income†**			
$60,000 or above	385 (28.88%)	–	–
$40,000–$59,999	304 (22.81%)	–	–
$20,000–$39,999	383 (28.73%)	–	–
$19,999 or below	261 (19.58%)	–	–
**Resource**, ***mean*** **(*SD*)**			
			0.698
T2 Self-efficacy	–	15.82 ± 3.31	
T3 Self-efficacy	–	15.85 ± 2.73	
			<0.001
T2 Perceived social support	–	52.64 ± 11.70	
T3 Perceived social support	–	51.47 ± 9.49	
			0.006
T2 Financial capacity	–	24.63 ± 5.21	
T3 Financial capacity	–	24.35 ± 4.56	
**Clinical symptoms**, ***mean*** **(*SD*)**‡			
			<0.001
T1 Depressive symptoms	–	5.06 ± 4.66	
T2 Depressive symptoms	–	5.94 ± 5.27	
T3 Depressive symptoms§	–	6.37 ± 5.13	
			<0.001
T1 Anxiety symptoms	–	4.89 ± 4.77	
T2 Anxiety symptoms	–	5.38 ± 4.98	
T3 Anxiety symptoms§	–	5.75 ± 4.98	
**Probable psychiatric condition¶ ||**			
T1 Probable depression	240 (18.00%)	–	–
T2 Probable depression	292 (21.91%)	–	–
T3 Probable depression§	226 (24.94%)	–	–
T1 Probable anxiety	236 (17.70%)	–	–
T2 Probable anxiety	238 (17.85%)	–	–
T3 Probable anxiety§	172 (18.98%)	–	–
**Resource change patterns in self-efficacy**			
High-High (Persistent high resources)	635 (47.64%)	–	–
High-Low (Resource loss)	137 (10.28%)	–	–
Low-High (Resource gain)	178 (13.35%)	–	–
Low-Low (Persistent low resources)	383 (28.73%)	–	–
**Resource change patterns in perceived social support**			
High-High (Persistent high resources)	545 (40.89%)	–	–
High-Low (Resource loss)	139 (10.43%)	–	–
Low-High (Resource gain)	161 (12.08%)	–	–
Low-Low (Persistent low resources)	488 (36.61%)	–	–
**Resource change patterns in financial capacity**			
High-High (Persistent high resources)	592 (44.41%)	–	–
High-Low (Resource loss)	84 (6.30%)	–	–
Low-High (Resource gain)	208 (15.60%)	–	–
Low-Low (Persistent low resources)	449 (33.68%)	–	–
**Trajectories of depressive symptoms**			
Resilience	989 (74.19%)	–	–
Recovery	112 (8.40%)	–	–
Delayed distress	96 (7.20%)	–	–
Chronic distress	136 (10.20%)	–	–
**Trajectories of anxiety symptoms**			
Resilience	965 (72.39%)	–	–
Recovery	159 (11.93%)	–	–
Delayed distress	98 (7.35%)	–	–
Chronic distress	111 (8.33%)	–	–

“*n*” indicates the number of respondents; “%” indicates the proportion of respondents; “*Mean*” and “*SD*” indicate the mean score and standard deviation; “*p*” indicates the significance of repeated-measures comparison between timepoints.

†US$1≈HK$7.80.

‡The repeated-measures comparisons for clinical symptoms were done based on the *n* = 906 sample. Bonferroni-corrected post-hoc tests for the repeated measures comparison showed that scores for depressive symptoms at all three timepoints were different from one another (T1 vs. T2: *p*(adjusted) < 0.001; T2 vs. T3: *p*(adjusted) < 0.001; T1 vs. T3: *p*(adjusted) = 0.029), whereas only scores for anxiety symptoms at T1 were different from T2/T3 (T1 vs. T2: *p*(adjusted) < 0.001; T2 vs. T3: *p*(adjusted) = 0.076; T1 vs. T3: *p*(adjusted) < 0.001).

¶Probable depression was indicated by a score of 10 or above on the 9-item Patient Health Questionnaire (PHQ-9); probable anxiety was indicated by a score of 10 or above on the 7-item Generalized Anxiety Disorder scale (GAD-7).

**||** Based on pair-wise McNemar’s tests with Bonferroni corrections, the proportion of respondents with probable depression continually increased over the three timepoints (T1 vs. T2: *p*(adjusted) = 0.004; T2 vs. T3: *p*(adjusted) = 0.027; T1 vs. T3: *p*(adjusted) < 0.001), whereas the proportion of respondents with probable anxiety remained constant over the three timepoints (T1 vs. T2: *p*(adjusted) > 0.999; T2 vs. T3: *p*(adjusted) = 0.422; T1 vs. T3: *p*(adjusted) = 0.686). (Pair-wise McNemar’s tests involving T3 data were conducted on the *n* = 906 sample).

At different time points, the sample-level mean scores for depressive symptoms were 5.06 (*SD* = 4.66) (T1), 5.94 (*SD* = 5.27) (T2), and 6.37 (*SD* = 5.13) (T3), showing an increase over the three timepoints (*p*s ≤ 0.029). Anxiety symptoms were 4.89 (*SD* = 4.77) (T1), 5.38 (*SD* = 4.98) (T2), and 5.75 (*SD* = 4.98) (T3), with the increase from T1 to T2 being significant (*p*s < 0.001) but the symptom level remained constant from T2 to T3 (*p* = 0.076). The proportions of respondents with probable depression were 18.00% (T1), 21.91% (T2), and 24.94% (T3), with a steady increase over the three timepoints (*p*s ≤ 0.027), and those with probable anxiety were 17.70% (T1), 17.85% (T2), and 18.98% (T3), which remained constant (*p* ≥ 0.422).

### Unconditional GMM Models

**Fig. 1:**
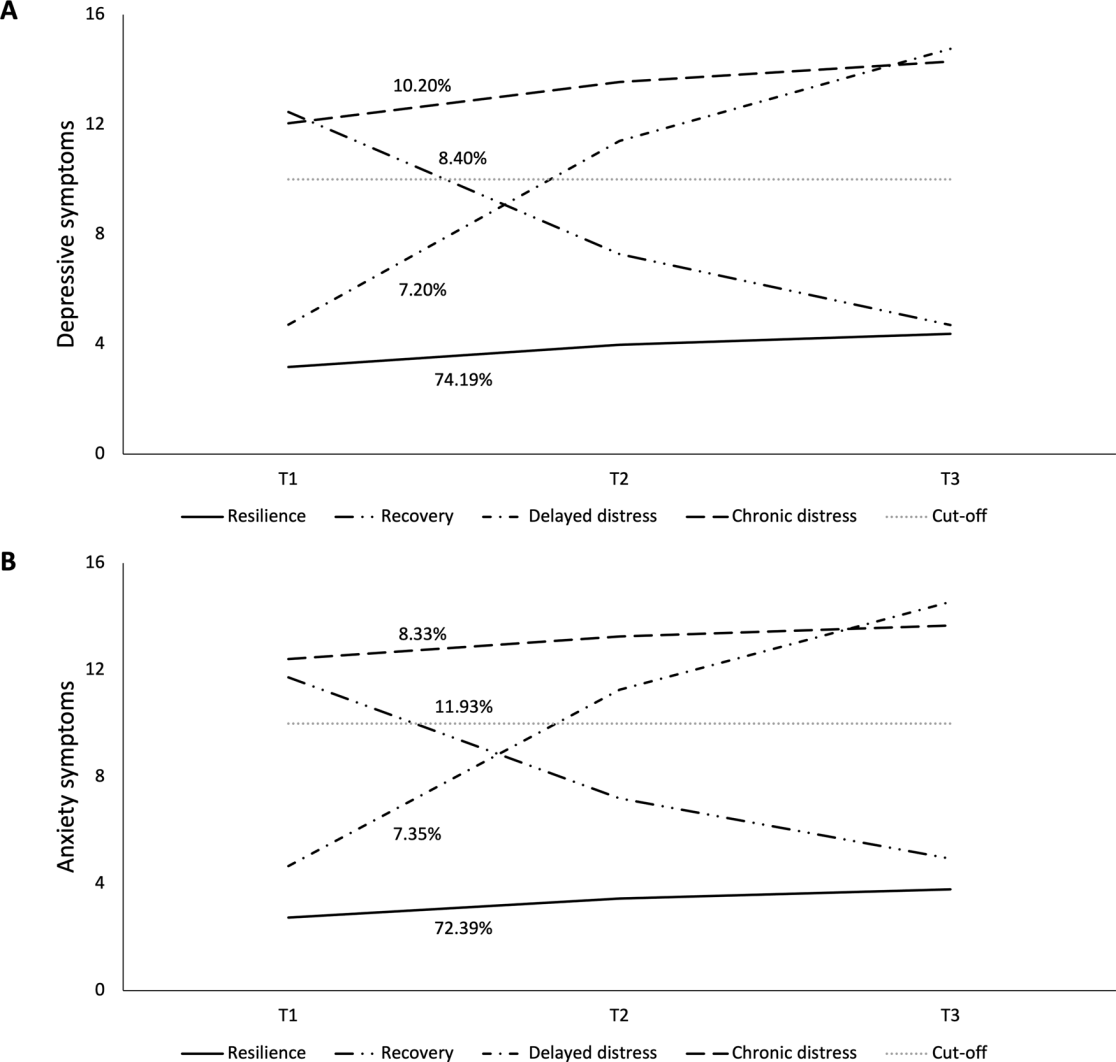
Trajectories of depressive and anxiety symptoms. Note. Probable depression was indicated by a score of 10 or above on the 9-item Patient Health Questionnaire (PHQ-9); probable anxiety was indicated by a score of 10 or above on the 7-item Generalized Anxiety Disorder scale (GAD-7).

We tested 1–5 class trajectory solutions of depressive and anxiety symptoms individually. Model fit indices are summarized in Supplementary Material 2. Results suggested that 4-class solutions demonstrated good model fit and adequate entropy. The proportion of each class is summarized in Table 1. Figure 1 depicts the 4-class trajectory models for depressive symptoms and for anxiety symptoms.

#### Depressive symptom trajectories

The *resilience* class had the highest prevalence (74.19%), characterized by a low intercept (*b* = 3.173, *SE* = 0.173, *p* < 0.001) and a significant positive but gentle slope (*b* = 0.800, *SE* = 0.132, *p* < 0.001). The *chronic distress* class (10.20%) with a high intercept (*b* = 12.041, *SE* = 0.601, *p* < 0.001) demonstrated a significant positive slope (*b* = 1.505, *SE* = 0.675, *p* = 0.026), the *recovery* class (8.40%) a high intercept (*b* = 12.454, *SE* = 0.769, *p* < 0.001) and a significant negative slope (*b* = –5.173, *SE* = 0.761, *p* < 0.001), and the *delayed distress* class (7.20%) a low intercept (*b* = 4.708, *SE* = 0.762, *p* < 0.001) and a significant positive slope (*b* = 6.697, *SE* = 0.623, *p* < 0.001).

#### Anxiety symptom trajectories

The *resilience* class had the highest prevalence (72.39%), characterized by a low intercept (*b* = 2.731, *SE* = 0.169, *p* < 0.001) and a significant positive but gentle slope (*b* = 0.708, *SE* = 0.109, *p* < 0.001). This was followed by the *recovery* class (11.93%), with a high intercept (*b* = 11.720, *SE* = 0.627, *p* < 0.001) and a significant negative slope (*b* = –4.518, *SE* = 0.558, *p* < .001), the *chronic distress* class (8.33%), with a high intercept (*b* = 12.408, *SE* = 0.677, *p* < 0.001) and a non-significant slope (*b* = 0.843, *SE* = 0.502, *p* = .093), and finally, the *delayed distress* class (7.35%), with a low intercept (*b* = 4.665, *SE* = 0.488, *p* < 0.001) and a significant positive slope (*b* = 6.594, *SE* = 0.598, *p* < 0.001).

#### Convergence across depressive symptom and anxiety symptom trajectories

In terms of the overlap in trajectory membership for depressive and anxiety symptoms, a non-random distribution was observed (χ^2^(9) = 1152.16, *p* < 0.001). More specifically, the majority of respondents in resilience class for depressive symptoms were also in the same class for anxiety symptoms (88.68%). However, the proportion of overlap was much smaller for respondents in the recovery (58.93%), the delayed distress (54.17%), and chronic distress (57.35%) classes. Table 2 summarizes the number of respondents showing different combinations of depressive and anxiety symptom trajectories.

**Table 2:** Distribution of respondents showing different combinations of depressive and anxiety symptom trajectories.

	Depressive symptom trajectories			
	Resilience (*n* = 989)	Recovery (*n* = 112)	Delayed distress (*n* = 96)	Chronic distress (*n* = 136)
**Anxiety symptom trajectories**				
Resilience	877 (88.68%)	34 (30.36%)	35 (36.46%)	19 (13.97%)
Recovery	64 (6.47%)	66 (58.93%)	2 (2.08%)	27 (19.85%)
Delayed distress	32 (3.24%)	2 (1.79%)	52 (54.17%)	12 (8.82%)
Chronic distress	16 (1.62%)	10 (8.93%)	7 (7.29%)	78 (57.35%)

### Conditional GMM Models

Tables 3 and 4 summarize the detailed odds ratios (ORs) and 95% confidence intervals (CIs) and the associations between trajectories and demographics.

**Table 3:** Conditional growth mixture modeling (GMM) results of depressive symptom trajectories.

		Reference class			
Class		Chronic distress	Delayed distress	Recovery	Resilience
	Covariates	OR (95% CI)	OR (95% CI)	OR (95% CI)	OR (95% CI)
Chronic distress					
*Demographics*					
Gender	Male	–	1	1	1
	Female	–	1.176 (0.476, 2.905)	0.664 (0.279, 1.584)	1.411 (0.759, 2.622)
Age	55 or above	–	1	1	1
	45–54	–	3.500 (0.119, 103.211)	7.198 (0.491, 105.425)	4.841 (0.451, 51.924)
	35–44	–	3.778 (0.161, 88.814)	**13.043 (1.042, 163.319)**	**16.508 (1.665, 163.707)**
	25–34	–	2.993 (0.123, 73.089)	**13.403 (1.033, 173.854)**	**19.207 (1.841, 200.429)**
	15–24	–	1.308 (0.051, 33.299)	**15.331 (1.061, 221.491)**	**25.761 (2.310, 287.275)**
Education	Tertiary or above	–	1	1	1
	Secondary or below	–	1.113 (0.392, 3.157)	0.671 (0.243, 1.849)	1.732 (0.829, 3.617)
Marital status	Married	–	1	1	1
	Single/divorced/widowed	–	0.951 (0.273, 3.317)	0.758 (0.274, 2.101)	1.188 (0.545, 2.590)
Employment	Employed	–	1	1	1
	Dependent	–	2.820 (0.753, 10.568)	1.073 (0.330, 3.492)	0.738 (0.293, 1.863)
	Unemployed	–	2.001 (0.285, 14.073)	0.519 (0.121, 2.223)	1.382 (0.431, 4.424)
Income†	$60,000 or above	–	1	1	1
	$40,000–$59,999	–	1.295 (0.347, 4.828)	0.906 (0.274, 2.992)	0.511 (0.211, 1.233)
	$20,000–$39,999	–	0.559 (0.170, 1.839)	0.726 (0.216, 2.438)	**0.300 (0.125, 0.718)**
	$19,999 or below	–	2.189 (0.375, 12.785)	0.931 (0.233, 3.721)	0.486 (0.166, 1.426)
*Resources*					
Personal	Low-Low (Persistent low)‡	–	2.874 (0.824, 10.032)	**5.625 (1.711, 18.492)**	**5.294 (2.106, 13.308)**
	High-Low (Loss)‡	–	0.239 (0.031, 1.836)	0.849 (0.122, 5.925)	1.064 (0.196, 5.791)
	Low-High (Gain)§	–	4.088 (0.558, 29.937)	1.276 (0.350, 4.656)	0.787 (0.381, 1.627)
	High-High (Persistent high)§	–	0.348 (0.100, 1.214)	**0.178 (0.054, 0.584)**	**0.189 (0.075, 0.475)**
Social	Low-Low (Persistent low)‡	–	1.230 (0.308, 4.909)	3.161 (0.934, 10.704)	**7.399 (2.757, 19.856)**
	High-Low (Loss)‡	–	11.923 (0.592, 240.254)	**9.521 (1.530, 59.247)**	**6.854 (2.097, 22.406)**
	Low-High (Gain)§	–	1.176 (0.273, 5.074)	0.547 (0.135, 2.216)	**0.338 (0.117, 0.979)**
	High-High (Persistent high)§	–	0.813 (0.204, 3.244)	0.316 (0.093, 1.071)	**0.135 (0.050, 0.363)**
Financial	Low-Low (Persistent low)‡	–	1.479 (0.407, 5.377)	**5.064 (1.437, 17.849)**	**6.022 (2.290, 15.838)**
	High-Low (Loss)‡	–	2.446 (0.358, 16.685)	3.677 (0.654, 20.659)	**5.739 (1.493, 22.054)**
	Low-High (Gain)§	–	1.226 (0.323, 4.653)	0.379 (0.117, 1.232)	**0.430 (0.186, 0.997)**
	High-High (Persistent high)§	–	0.676 (0.186, 2.459)	**0.197 (0.056, 0.696)**	**0.166 (0.063, 0.437)**
Delayed distress					
*Demographics*					
Gender	Male	1	–	1	1
	Female	0.850 (0.344, 2.101)	–	0.565 (0.245, 1.304)	1.200 (0.580, 2.483)
Age	55 or above	1	–	1	1
	45–54	0.286 (0.010, 8.424)	–	2.056 (0.226, 18.696)	1.383 (0.173, 11.027)
	35–44	0.265 (0.011, 6.221)	–	3.452 (0.497, 24.001)	4.369 (0.659, 28.967)
	25–34	0.334 (0.014, 8.159)	–	4.478 (0.648, 30.932)	6.417 (0.960, 42.903)
	15–24	0.765 (0.030, 19.464)	–	**11.721 (1.581, 86.882)**	**19.696 (2.901, 133.697)**
Education	Tertiary or above	1	–	1	1
	Secondary or below	0.899 (0.317, 2.550)	–	0.603 (0.229, 1.586)	1.556 (0.678, 3.571)
Marital status	Married	1	–	1	1
	Single/divorced/widowed	1.052 (0.302, 3.669)	–	0.798 (0.270, 2.353)	1.250 (0.457, 3.416)
Employment	Employed	1	–	1	1
	Dependent	0.355 (0.095, 1.329)	–	0.381 (0.125, 1.156)	**0.262 (0.094, 0.727)**
	Unemployed	0.500 (0.071, 3.513)	–	0.259 (0.046, 1.452)	0.690 (0.128, 3.729)
Income†	$60,000 or above	1	–	1	1
	$40,000–$59,999	0.772 (0.207, 2.880)	–	0.700 (0.215, 2.279)	0.394 (0.138, 1.123)
	$20,000–$39,999	1.788 (0.544, 5.876)	–	1.297 (0.419, 4.020)	0.536 (0.206, 1.392)
	$19,999 or below	0.457 (0.078, 2.668)	–	0.425 (0.085, 2.140)	0.222 (0.048, 1.025)
*Resources*					
Personal	Low-Low (Persistent low)‡	0.348 (0.100, 1.214)	–	1.957 (0.716, 5.351)	1.842 (0.776, 4.372)
	High-Low (Loss)‡	4.188 (0.545, 32.198)	–	**3.554 (1.082, 11.679)**	**4.457 (1.513, 13.130)**
	Low-High (Gain)§	0.245 (0.033, 1.791)	–	0.312 (0.039, 2.486)	0.193 (0.028, 1.343)
	High-High (Persistent high)§	2.874 (0.824, 10.032)	–	0.511 (0.187, 1.397)	0.543 (0.229, 1.289)
Social	Low-Low (Persistent low)‡	0.813 (0.204, 3.244)	–	2.570 (0.931, 7.092)	**6.014 (2.438, 14.838)**
	High-Low (Loss)‡	0.084 (0.004, 1.690)	–	0.799 (0.046, 13.937)	0.575 (0.039, 8.519)
	Low-High (Gain)§	0.850 (0.197, 3.667)	–	0.465 (0.131, 1.648)	**0.287 (0.096, 0.861)**
	High-High (Persistent high)§	1.230 (0.308, 4.909)	–	0.389 (0.141, 1.074)	**0.166 (0.067, 0.410)**
Financial	Low-Low (Persistent low)‡	0.676 (0.186, 2.459)	–	**3.424 (1.204, 9.741)**	**4.072 (1.693, 9.794)**
	High-Low (Loss)‡	0.409 (0.060, 2.789)	–	1.503 (0.284, 7.955)	2.347 (0.503, 10.941)
	Low-High (Gain)§	0.816 (0.215, 3.095)	–	0.309 (0.091, 1.053)	0.351 (0.118, 1.047)
	High-High (Persistent high)§	1.479 (0.407, 5.377)	–	**0.292 (0.103, 0.831)**	**0.246 (0.102, 0.591)**
Recovery					
*Demographics*					
Gender	Male	1	1	–	1
	Female	1.505 (0.631, 3.588)	1.770 (0.767, 4.084)	–	**2.123 (1.182, 3.814)**
Age	55 or above	1	1	–	1
	45–54	0.139 (0.009, 2.035)	0.486 (0.053, 4.421)	–	0.672 (0.238, 1.898)
	35–44	**0.077 (0.006, 0.960)**	0.290 (0.042, 2.014)	–	1.266 (0.529, 3.027)
	25–34	**0.075 (0.006, 0.968)**	0.223 (0.032, 1.542)	–	1.433 (0.607, 3.381)
	15–24	**0.065 (0.005, 0.942)**	**0.085 (0.012, 0.632)**	–	1.680 (0.626, 4.511)
Education	Tertiary or above	1	1	–	1
	Secondary or below	1.491 (0.541, 4.112)	1.659 (0.631, 4.367)	–	**2.583 (1.296, 5.149)**
Marital status	Married	1	1	–	1
	Single/divorced/widowed	1.319 (0.476, 3.654)	1.254 (0.425, 3.700)	–	1.567 (0.826, 2.975)
Employment	Employed	1	1	–	1
	Dependent	0.932 (0.286, 3.031)	2.627 (0.865, 7.979)	–	0.688 (0.344, 1.375)
	Unemployed	1.928 (0.450, 8.258)	3.858 (0.689, 21.615)	–	**2.663 (1.008, 7.033)**
Income†	$60,000 or above	1	1	–	1
	$40,000–$59,999	1.104 (0.334, 3.647)	1.429 (0.439, 4.657)	–	0.564 (0.259, 1.225)
	$20,000–$39,999	1.378 (0.410, 4.629)	0.771 (0.249, 2.389)	–	**0.413 (0.184, 0.929)**
	$19,999 or below	1.074 (0.269, 4.289)	2.351 (0.467, 11.823)	–	0.522 (0.223, 1.223)
*Resources*					
Personal	Low-Low (Persistent low)‡	**0.178 (0.054, 0.584)**	0.511 (0.187, 1.397)	–	0.941 (0.461, 1.920)
	High-Low (Loss)‡	1.178 (0.169, 8.226)	**0.281 (0.086, 0.925)**	–	1.254 (0.548, 2.870)
	Low-High (Gain)§	0.784 (0.215, 2.860)	3.204 (0.402, 25.525)	–	0.617 (0.206, 1.849)
	High-High (Persistent high)§	**5.625 (1.711, 18.492)**	1.957 (0.716, 5.351)	–	1.062 (0.521, 2.167)
Social	Low-Low (Persistent low)‡	0.316 (0.093, 1.071)	0.389 (0.141, 1.074)	–	**2.341 (1.222, 4.484)**
	High-Low (Loss)‡	**0.105 (0.017, 0.654)**	1.252 (0.072, 21.856)	–	0.720 (0.200, 2.593)
	Low-High (Gain)§	1.827 (0.451, 7.397)	2.149 (0.607, 7.609)	–	0.617 (0.257, 1.486)
	High-High (Persistent high)§	3.161 (0.934, 10.704)	2.570 (0.931, 7.092)	–	**0.427 (0.223, 0.818)**
Financial	Low-Low (Persistent low)‡	**0.197 (0.056, 0.696)**	**0.292 (0.103, 0.831)**	–	1.189 (0.563, 2.514)
	High-Low (Loss)‡	0.272 (0.048, 1.528)	0.665 (0.126, 3.520)	–	1.561 (0.555, 4.389)
	Low-High (Gain)§	2.636 (0.811, 8.563)	3.232 (0.950, 10.998)	–	1.134 (0.497, 2.592)
	High-High (Persistent high)§	**5.064 (1.437, 17.849)**	**3.424 (1.204, 9.741)**	–	0.841 (0.398, 1.778)
Resilience					
*Demographics*					
Gender	Male	1	1	1	–
	Female	0.709 (0.381, 1.317)	0.833 (0.403, 1.725)	**0.471 (0.262, 0.846)**	–
Age	55 or above	1	1	1	–
	45–54	0.207 (0.019, 2.216)	0.723 (0.091, 5.766)	1.487 (0.527, 4.196)	–
	35–44	**0.061 (0.006, 0.601)**	0.229 (0.035, 1.517)	0.790 (0.330, 1.889)	–
	25–34	**0.052 (0.005, 0.543)**	0.156 (0.023, 1.042)	0.698 (0.296, 1.646)	–
	15–24	**0.039 (0.003, 0.433)**	**0.051 (0.007, 0.345)**	0.595 (0.222, 1.598)	–
Education	Tertiary or above	1	1	1	–
	Secondary or below	0.577 (0.276, 1.206)	0.642 (0.280, 1.474)	**0.387 (0.194, 0.772)**	–
Marital status	Married	1	1	1	–
	Single/divorced/widowed	0.842 (0.386, 1.834)	0.800 (0.293, 2.187)	0.638 (0.336, 1.211)	–
Employment	Employed	1	1	1	–
	Dependent	1.355 (0.537, 3.419)	**3.820 (1.375, 10.613)**	1.454 (0.727, 2.908)	–
	Unemployed	0.724 (0.226, 2.318)	1.449 (0.268, 7.825)	**0.376 (0.142, 0.992)**	–
Income†	$60,000 or above	1	1	1	–
	$40,000–$59,999	1.959 (0.811, 4.732)	2.536 (0.890, 7.226)	1.774 (0.817, 3.855)	–
	$20,000–$39,999	**3.336 (1.392, 7.991)**	1.866 (0.718, 4.849)	**2.421 (1.077, 5.442)**	–
	$19,999 or below	2.056 (0.701, 6.026)	4.500 (0.976, 20.748)	1.915 (0.818, 4.483)	–
*Resources*					
Personal	Low-Low (Persistent low)‡	**0.189 (0.075, 0.475)**	0.543 (0.229, 1.289)	1.062 (0.521, 2.167)	–
	High-Low (Loss)‡	0.940 (0.173, 5.113)	**0.224 (0.076, 0.661)**	0.797 (0.348, 1.825)	–
	Low-High (Gain)§	1.271 (0.615, 2.626)	5.194 (0.744, 36.249)	1.621 (0.541, 4.858)	–
	High-High (Persistent high)§	**5.294 (2.106, 13.308)**	1.842 (0.776, 4.372)	0.941 (0.461, 1.920)	–
Social	Low-Low (Persistent low)‡	**0.135 (0.050, 0.363)**	**0.166 (0.067, 0.410)**	**0.427 (0.223, 0.818)**	–
	High-Low (Loss)‡	**0.146 (0.045, 0.477)**	1.740 (0.117, 25.782)	1.389 (0.386, 5.003)	–
	Low-High (Gain)§	**2.959 (1.022, 8.572)**	**3.481 (1.161, 10.436)**	1.620 (0.673, 3.897)	–
	High-High (Persistent high)§	**7.399 (2.757, 19.856)**	**6.014 (2.438, 14.838)**	**2.341 (1.222, 4.484)**	–
Financial	Low-Low (Persistent low)‡	**0.166 (0.063, 0.437)**	**0.246 (0.102, 0.591)**	0.841 (0.398, 1.778)	–
	High-Low (Loss)‡	**0.174 (0.045, 0.670)**	0.426 (0.091, 1.987)	0.641 (0.228, 1.801)	–
	Low-High (Gain)§	**2.324 (1.003, 5.384)**	2.849 (0.955, 8.499)	0.882 (0.386, 2.014)	–
	High-High (Persistent high)§	**6.022 (2.290, 15.838)**	**4.072 (1.693, 9.794)**	1.189 (0.563, 2.514)	–

Bold texts indicate significant results.

†US$1≈HK$7.80.

‡The “Low-Low (Persistent low)” and “High-Low (Loss)” groups were compared against the “High-High (Persistent high)” group.

§The “Low-High (Gain)” and “High-High (Persistent high)” groups were compared against the “Low-Low (Persistent low)” group.

**Table 4:** Conditional growth mixture modeling (GMM) results of anxiety symptom trajectories.

		Reference class			
Class		Chronic distress	Delayed distress	Recovery	Resilience
	Covariates	OR (95% CI)	OR (95% CI)	OR (95% CI)	OR (95% CI)
Chronic distress					
*Demographics*					
Gender	Male	–	1	1	1
	Female	–	0.792 (0.350, 1.793)	0.879 (0.412, 1.874)	1.574 (0.884, 2.804)
Age	55 or above	–	1	1	1
	45–54	–	1.171 (0.160, 8.592)	0.862 (0.158, 4.696)	0.891 (0.219, 3.620)
	35–44	–	2.191 (0.467, 10.280)	2.593 (0.667, 10.078)	**3.851 (1.350, 10.986)**
	25–34	–	1.443 (0.305, 6.834)	1.951 (0.488, 7.802)	**4.113 (1.370, 12.346)**
	15–24	–	0.757 (0.148, 3.876)	1.789 (0.387, 8.261)	**4.412 (1.298, 14.990)**
Education	Tertiary or above	–	1	1	1
	Secondary or below	–	0.479 (0.182, 1.261)	0.517 (0.207, 1.292)	0.805 (0.387, 1.671)
Marital status	Married	–	1	1	1
	Single/divorced/widowed	–	0.937 (0.333, 2.635)	0.778 (0.319, 1.897)	0.758 (0.383, 1.502)
Employment	Employed	–	1	1	1
	Dependent	–	**3.392 (1.111, 10.351)**	1.902 (0.661, 5.472)	1.203 (0.524, 2.759)
	Unemployed	–	4.343 (0.694, 27.178)	3.017 (0.663, 13.731)	2.114 (0.817, 5.469)
Income†	$60,000 or above	–	1	1	1
	$40,000–$59,999	–	2.466 (0.791, 7.687)	1.347 (0.491, 3.697)	0.815 (0.380, 1.746)
	$20,000–$39,999	–	1.187 (0.380, 3.701)	0.681 (0.238, 1.947)	**0.410 (0.175, 0.957)**
	$19,999 or below	–	2.668 (0.649, 10.972)	1.404 (0.396, 4.981)	0.693 (0.263, 1.826)
*Resources*					
Personal	Low-Low (Persistent low)‡	–	2.210 (0.733, 6.667)	2.287 (0.848, 6.171)	**4.830 (2.192, 10.643)**
	High-Low (Loss)‡	–	0.911 (0.216, 3.840)	2.519 (0.569, 11.155)	2.376 (0.803, 7.028)
	Low-High (Gain)§	–	0.932 (0.308, 2.816)	0.921 (0.349, 2.430)	0.781 (0.379, 1.612)
	High-High (Persistent high)§	–	0.452 (0.150, 1.365)	0.437 (0.162, 1.179)	**0.207 (0.094, 0.456)**
Social	Low-Low (Persistent low)‡	–	0.882 (0.269, 2.895)	2.321 (0.847, 6.366)	**5.700 (2.511, 12.938)**
	High-Low (Loss)‡	–	2.041 (0.335, 12.443)	3.317 (0.761, 14.466)	**3.076 (1.083, 8.736)**
	Low-High (Gain)§	–	0.654 (0.189, 2.263)	0.696 (0.200, 2.418)	0.380 (0.143, 1.012)
	High-High (Persistent high)§	–	1.133 (0.345, 3.719)	0.431 (0.157, 1.181)	**0.175 (0.077, 0.398)**
Financial	Low-Low (Persistent low)‡	–	1.637 (0.578, 4.636)	1.914 (0.679, 5.392)	**3.050 (1.344, 6.921)**
	High-Low (Loss)‡	–	4.123 (0.680, 25.007)	3.696 (0.747, 18.284)	**3.649 (1.134, 11.743)**
	Low-High (Gain)§	–	2.456 (0.696, 8.662)	1.431 (0.529, 3.870)	0.918 (0.461, 1.826)
	High-High (Persistent high)§	–	0.611 (0.216, 1.730)	0.523 (0.185, 1.472)	**0.328 (0.144, 0.744)**
Delayed distress					
*Demographics*					
Gender	Male	1	–	1	1
	Female	1.263 (0.558, 2.860)	–	1.110 (0.561, 2.198)	**1.988 (1.075, 3.678)**
Age	55 or above	1	–	1	1
	45–54	0.854 (0.116, 6.267)	–	0.736 (0.172, 3.151)	0.761 (0.200, 2.888)
	35–44	0.457 (0.097, 2.142)	–	1.184 (0.342, 4.102)	1.758 (0.566, 5.463)
	25–34	0.693 (0.146, 3.284)	–	1.352 (0.393, 4.652)	2.851 (0.924, 8.798)
	15–24	1.321 (0.258, 6.763)	–	2.363 (0.679, 8.223)	**5.827 (1.927, 17.619)**
Education	Tertiary or above	1	–	1	1
	Secondary or below	2.087 (0.793, 5.492)	–	1.079 (0.491, 2.369)	1.679 (0.831, 3.393)
Marital status	Married	1	–	1	1
	Single/divorced/widowed	1.068 (0.380, 3.003)	–	0.830 (0.341, 2.020)	0.810 (0.360, 1.822)
Employment	Employed	1	–	1	1
	Dependent	**0.295 (0.097, 0.900)**	–	0.561 (0.226, 1.395)	**0.355 (0.157, 0.802)**
	Unemployed	0.230 (0.037, 1.441)	–	0.695 (0.104, 4.619)	0.487 (0.089, 2.650)
Income†	$60,000 or above	1	–	1	1
	$40,000–$59,999	0.405 (0.130, 1.264)	–	0.546 (0.204, 1.460)	**0.330 (0.135, 0.806)**
	$20,000–$39,999	0.843 (0.270, 2.628)	–	0.574 (0.234, 1.408)	**0.345 (0.151, 0.789)**
	$19,999 or below	0.375 (0.091, 1.542)	–	0.526 (0.154, 1.801)	**0.260 (0.085, 0.791)**
*Resources*					
Personal	Low-Low (Persistent low)‡	0.452 (0.150, 1.365)	–	1.035 (0.454, 2.357)	**2.185 (1.026, 4.654)**
	High-Low (Loss)‡	1.098 (0.260, 4.628)	–	2.766 (0.821, 9.322)	2.609 (0.986, 6.899)
	Low-High (Gain)§	1.073 (0.355, 3.242)	–	0.989 (0.376, 2.599)	0.838 (0.338, 2.079)
	High-High (Persistent high)§	2.210 (0.733, 6.667)	–	0.966 (0.424, 2.201)	**0.458 (0.215, 0.974)**
Social	Low-Low (Persistent low)‡	1.133 (0.345, 3.719)	–	**2.631 (1.064, 6.508)**	**6.461 (2.819, 14.807)**
	High-Low (Loss)‡	0.490 (0.080, 2.986)	–	1.625 (0.322, 8.208)	1.507 (0.355, 6.393)
	Low-High (Gain)§	1.528 (0.442, 5.286)	–	1.063 (0.407, 2.781)	0.580 (0.256, 1.317)
	High-High (Persistent high)§	0.882 (0.269, 2.895)	–	**0.380 (0.154, 0.940)**	**0.155 (0.068, 0.355)**
Financial	Low-Low (Persistent low)‡	0.611 (0.216, 1.730)	–	1.169 (0.524, 2.609)	1.863 (0.933, 3.721)
	High-Low (Loss)‡	0.243 (0.040, 1.471)	–	0.896 (0.175, 4.599)	0.885 (0.197, 3.981)
	Low-High (Gain)§	0.407 (0.115, 1.436)	–	0.583 (0.183, 1.856)	0.374 (0.128, 1.088)
	High-High (Persistent high)§	1.637 (0.578, 4.636)	–	0.855 (0.383, 1.910)	0.537 (0.269, 1.072)
Recovery					
*Demographics*					
Gender	Male	1	1	–	1
	Female	1.138 (0.534, 2.426)	0.901 (0.455, 1.783)	–	**1.791 (1.095, 2.929)**
Age	55 or above	1	1	–	1
	45–54	1.160 (0.213, 6.319)	1.358 (0.317, 5.813)	–	1.033 (0.432, 2.474)
	35–44	0.386 (0.099, 1.499)	0.845 (0.244, 2.927)	–	1.485 (0.657, 3.355)
	25–34	0.513 (0.128, 2.050)	0.739 (0.215, 2.543)	–	2.108 (0.911, 4.878)
	15–24	0.559 (0.121, 2.582)	0.423 (0.122, 1.473)	–	2.466 (0.992, 6.130)
Education	Tertiary or above	1	1	–	1
	Secondary or below	1.935 (0.774, 4.833)	0.927 (0.422, 2.036)	–	1.557 (0.872, 2.781)
Marital status	Married	1	1	–	1
	Single/divorced/widowed	1.286 (0.527, 3.137)	1.204 (0.495, 2.931)	–	0.975 (0.536, 1.774)
Employment	Employed	1	1	–	1
	Dependent	0.526 (0.183, 1.512)	1.783 (0.717, 4.434)	–	0.632 (0.328, 1.218)
	Unemployed	0.331 (0.073, 1.509)	1.440 (0.217, 9.572)	–	0.701 (0.202, 2.425)
Income†	$60,000 or above	1	1	–	1
	$40,000–$59,999	0.742 (0.271, 2.037)	1.831 (0.685, 4.893)	–	0.605 (0.309, 1.184)
	$20,000–$39,999	1.468 (0.514, 4.193)	1.742 (0.710, 4.271)	–	0.601 (0.317, 1.140)
	$19,999 or below	0.712 (0.201, 2.527)	1.900 (0.555, 6.501)	–	0.494 (0.208, 1.171)
*Resources*					
Personal	Low-Low (Persistent low)‡	0.437 (0.162, 1.179)	0.966 (0.424, 2.201)	–	**2.112 (1.196, 3.727)**
	High-Low (Loss)‡	0.397 (0.090, 1.757)	0.362 (0.107, 1.218)	–	0.943 (0.357, 2.491)
	Low-High (Gain)§	1.085 (0.412, 2.861)	1.011 (0.385, 2.659)	–	0.848 (0.426, 1.688)
	High-High (Persistent high)§	2.287 (0.848, 6.171)	1.035 (0.454, 2.357)	–	**0.474 (0.268, 0.836)**
Social	Low-Low (Persistent low)‡	0.431 (0.157, 1.181)	**0.380 (0.154, 0.940)**	–	**2.455 (1.404, 4.294)**
	High-Low (Loss)‡	0.301 (0.069, 1.315)	0.615 (0.122, 3.108)	–	0.927 (0.348, 2.473)
	Low-High (Gain)§	1.437 (0.414, 4.993)	0.940 (0.360, 2.459)	–	0.546 (0.256, 1.162)
	High-High (Persistent high)§	2.321 (0.847, 6.366)	**2.631 (1.064, 6.508)**	–	**0.407 (0.233, 0.712)**
Financial	Low-Low (Persistent low)‡	0.523 (0.185, 1.472)	0.855 (0.383, 1.910)	–	1.594 (0.854, 2.975)
	High-Low (Loss)‡	0.271 (0.055, 1.339)	1.116 (0.217, 5.724)	–	0.987 (0.326, 2.989)
	Low-High (Gain)§	0.699 (0.258, 1.889)	1.716 (0.539, 5.465)	–	0.641 (0.304, 1.353)
	High-High (Persistent high)§	1.914 (0.679, 5.392)	1.169 (0.524, 2.609)	–	0.627 (0.336, 1.171)
Resilience					
*Demographics*					
Gender	Male	1	1	1	–
	Female	0.635 (0.357, 1.131)	**0.503 (0.272, 0.930)**	**0.558 (0.341, 0.913)**	–
Age	55 or above	1	1	1	–
	45–54	1.122 (0.276, 4.561)	1.314 (0.346, 4.989)	0.968 (0.404, 2.317)	–
	35–44	**0.260 (0.091, 0.741)**	0.569 (0.183, 1.768)	0.673 (0.298, 1.521)	–
	25–34	**0.243 (0.081, 0.730)**	0.351 (0.114, 1.083)	0.474 (0.205, 1.098)	–
	15–24	**0.227 (0.067, 0.770)**	**0.172 (0.057, 0.519)**	0.405 (0.163, 1.008)	–
Education	Tertiary or above	1	1	1	–
	Secondary or below	1.243 (0.598, 2.581)	0.596 (0.295, 1.203)	0.642 (0.360, 1.147)	–
Marital status	Married	1	1	1	–
	Single/divorced/widowed	1.318 (0.666, 2.610)	1.235 (0.549, 2.778)	1.025 (0.564, 1.865)	–
Employment	Employed	1	1	1	–
	Dependent	0.831 (0.362, 1.907)	**2.820 (1.247, 6.376)**	1.581 (0.821, 3.046)	–
	Unemployed	0.473 (0.183, 1.223)	2.054 (0.377, 11.184)	1.427 (0.412, 4.938)	–
Income†	$60,000 or above	1	1	1	–
	$40,000–$59,999	1.227 (0.573, 2.630)	**3.027 (1.241, 7.380)**	1.653 (0.845, 3.236)	–
	$20,000–$39,999	**2.442 (1.045, 5.704)**	**2.898 (1.267, 6.629)**	1.664 (0.877, 3.157)	–
	$19,999 or below	1.442 (0.548, 3.799)	**3.848 (1.265, 11.705)**	2.025 (0.854, 4.800)	–
*Resources*					
Personal	Low-Low (Persistent low)‡	**0.207 (0.094, 0.456)**	**0.458 (0.215, 0.974)**	**0.474 (0.268, 0.836)**	–
	High-Low (Loss)‡	0.421 (0.142, 1.245)	0.383 (0.145, 1.014)	1.060 (0.401, 2.801)	–
	Low-High (Gain)§	1.280 (0.620, 2.642)	1.193 (0.481, 2.960)	1.180 (0.592, 2.350)	–
	High-High (Persistent high)§	**4.830 (2.192, 10.643)**	**2.185 (1.026, 4.654)**	**2.112 (1.196, 3.727)**	–
Social	Low-Low (Persistent low)‡	**0.175 (0.077, 0.398)**	**0.155 (0.068, 0.355)**	**0.407 (0.233, 0.712)**	–
	High-Low (Loss)‡	**0.325 (0.114, 0.924)**	0.664 (0.156, 2.816)	1.079 (0.404, 2.876)	–
	Low-High (Gain)§	2.633 (0.988, 7.017)	1.723 (0.759, 3.911)	1.833 (0.861, 3.903)	–
	High-High (Persistent high)§	**5.700 (2.511, 12.938)**	**6.461 (2.819, 14.807)**	**2.455 (1.404, 4.294)**	–
Financial	Low-Low (Persistent low)‡	**0.328 (0.144, 0.744)**	0.537 (0.269, 1.072)	0.627 (0.336, 1.171)	–
	High-Low (Loss)‡	**0.274 (0.085, 0.882)**	1.130 (0.251, 5.083)	1.013 (0.335, 3.066)	–
	Low-High (Gain)§	1.089 (0.548, 2.168)	2.676 (0.919, 7.791)	1.559 (0.739, 3.288)	–
	High-High (Persistent high)§	**3.050 (1.344, 6.921)**	1.863 (0.933, 3.721)	1.594 (0.854, 2.975)	–

Bold texts indicate significant results.

†US$1≈HK$7.80.

‡The “Low-Low (Persistent low)” and “High-Low (Loss)” groups were compared against the “High-High (Persistent high)” group.

§The “Low-High (Gain)” and “High-High (Persistent high)” groups were compared against the “Low-Low (Persistent low)” group.

#### Depressive symptom trajectories

Relative to the *resilience* group, individuals in the chronic distress group were characterized by higher odds of resource loss and lower odds of resource gain in perceived social support and financial capacity. Individuals in the delayed distress group were characterized by higher odds of resource loss in self-efficacy and lower odds of resource gain in perceived social support. Relative to the *chronic distress* group, individuals in the recovery group were characterized by lower odds of resource loss in perceived social support. Additionally, persistent low resources in perceived social support distinguished non-resilience (i.e., chronic distress, delayed distress, recovery) from resilience trajectories, whereas persistent high resources in self-efficacy and financial capacity distinguished resilience/recovery from chronic distress. Detailed statistical results are summarized in Table 3.

#### Anxiety symptom trajectories

Relative to the *resilience* group, individuals in the chronic distress group were characterized by resource loss in perceived social support and financial capacity. Resource changes in delayed distress or recovery groups were not different from resource changes in resilience or chronic distress groups. Persistent low self-efficacy and perceived social support distinguished non-resilience (i.e., chronic distress, delayed distress, recovery) from resilience trajectories. Detailed statistical results are summarized in Table 4.

## Discussion

This is a three-wave prospective study on a population-representative sample that adopted growth mixture modeling (GMM) to test the associations between different patterns of resource changes and psychological outcome trajectories amid the COVID-19 pandemic. Consistent with *Hypothesis 1*, the majority of respondents displayed a resilience trajectory in the first two years following the outbreak, with the remaining participants divided among recovery, delayed distress, and chronic distress trajectories. Resource loss (and/or a lack of resource gain) in financial capacity characterized chronic distress in both depressive and anxiety symptoms. Resource loss in self-efficacy characterized delayed distress in depressive symptoms. Resource loss (and/or a lack of resource gain) in perceived social support was associated with chronic distress in depressive/anxiety symptoms and delayed distress in depressive symptoms, whereas a lack of resource loss was associated with recovery from depressive symptoms. Patterns of resource changes partially overlapped with outcome trajectories in shape (*Hypotheses 2* and *3*).

### Outcome trajectories and resource changes

That the majority of people exhibited a resilient trajectory during COVID-19 (>70%) was consistent with trajectories previously identified in China [5], Poland [6], Israel [7], and other countries. Taken together, these findings support the generalizability of the resilience literature to the COVID-19 pandemic [4]. Additionally, the current findings (from February 2020 through February 2022) could be validly applied to understand psychological adaptation as COVID-19 shifted from acute health threat to a constellation of long-lasting and multifaceted stressors [58]. This large-scale disaster has turned out to show significant impact beyond the public health sector, including economic, social, and environmental domains [59]. The rising trend of clinically significant depressive and anxiety symptoms in our study could reflect the increasing challenges people face in the post-COVID-19 era.

This study provided some of the first evidence on changes in different dimensions of resources and the associations of the changes with trajectories of psychopathology and resilience across the first two years of the COVID-19 pandemic, while similar investigations have been relatively scarce [60]. The majority of respondents displayed either persistent high (40–50%) or persistent low (~30%) personal, social, and/or financial resources, whereas the remaining 20% of the sample was divided between resource loss (10%) and gain (10%). The distribution was fairly comparable across the three types of resources. The 10% respondents with resource loss was comparable to the rate in a previous study reporting deteriorations in perceived health, household economic situation, and tension among Israeli families [61]. This rate was nevertheless lower than the proportion of 20–30% people with losses in income, perceived control in future, entertainment, and interpersonal relationships among Chinese populations [23, 62, 63]. The discrepancy in the findings could be because subjective resources were assessed in the current study whereas more objective income reduction was assessed in previous studies.

### Trajectories as functions of changes in different resources

Within the current COVID-19 pandemic, trajectories of resilience and/or recovery, relative to chronic and/or delayed distress, were characterized by higher levels of coping resources [6, 7, 28, 56]. There is evidence showing that clinically significant symptoms were reported in conjunction with resource loss [23, 25–27]. Our study analyzed three key types of resources (i.e., personal, social, financial), and revealed their distinctive roles in promoting resilience and protecting against psychopathology.

Within the pandemic, individuals displaying clinically significant depressive and anxiety symptoms over time reported more economic difficulties than those displaying resilience [7], whereas cumulative economic hardship prospectively predicted greater psychological distress [29, 64]. Extending these preexisting findings, the current results showed how *changes*—loss and/or gain—in financial resources over time were associated with chronic distress and resilience trajectories, respectively, across depressive and anxiety symptoms. Depending on the direction, changes in financial resources could indicate persistent optimal or suboptimal conditions, but less as risk/protective factors, probably because economic vulnerability is deep-seated [64] and its psychological consequences are chronic rather than dynamic [65, 66], particularly under the widespread consequences of the Great Lockdown as the macroeconomic environment [67].

As an important element of coping within a health context, self-efficacy was found to be positively associated with self-reported mental health [68] and inversely associated with risks of depression [56, 69] within COVID-19. The current study revealed that loss but not gain in self-efficacy uniquely characterized delayed distress in depressive symptoms. Previous evidence similarly documented that individuals’ ability to retain their efficacy to cope under disasters importantly limited psychological distress [70], but enhanced perceptions of current selves did not exhibit additional protective effect [71]. Personal resources probably exhibit an indirect benefit on mental health, possibly through individuals’ proactive rectification of environmental demands [70].

Following disasters with more acute stressors, communal resources such as one’s trust and reciprocity within the community, social support, and city-level infrastructural support inversely predicted psychiatric symptoms [8, 72] and positively predicted the odds of resilience or recovery from clinically significant psychiatric symptoms [20, 56]. In the COVID-19 pandemic, perceived social support was concurrently associated with lower odds of probable depression/anxiety [73] and prospectively related to decreased distress [28]. Social support leads to higher well-being, because of both its direct positive effect and its buffering role against adverse consequences of stressors [74–76]. Our findings further showed that *changes* in social resources—both loss and gain—were highly sensitive to the four outcome trajectories, and could distinguish individuals with delayed distress from those with resilience, and those in recovery from those with chronic distress.

Taken together, our results, alongside previous findings, supported the COR theory that the process of stress adaptation is driven by resource gain and/or loss [11]. Gain or loss in resources has been suggested to play an additional role in shaping psychological adjustment than the specific level of resources at a given moment [11, 22]. Losses in personal, social, and/or material resources positively predicted psychiatric symptoms [24, 77, 78] and increased the odds of chronic or delayed distress [8, 21]. Notably, existing studies on resource changes and clinical trajectories tended to focus on resource loss [24, 77, 78], while gain, as well as no change, were less often studied as separate, predictive conditions. Relatedly, our results revealed that resource gain also plays a role in shaping outcome trajectories, although the effects might be more specific to depressive symptoms [14, 15]. Finally, the resource caravan passageways in the COR theory [14, 15] outlined how initial resource gain/loss could lead to gain/loss spirals. Distinctly different resources studied in the current investigation should therefore be understood in light of their formation of composite, non-mutually-exclusive elements in aid of adaptation.

### Limitations, strengths, and implications

This study has several limitations. First, depressive and anxiety symptoms were self-reported, and our findings could be strengthened by having a larger sample in order to include more respondents meeting the designated clinical cutoff. The current rates of probable depression and anxiety and their trajectories await cross-validation with clinical interviews/diagnoses in future studies. Future studies will also benefit from the inclusion of a PTSD-related measure, as a confounder and/or as an outcome. Second, we assessed subjective perceptions of personal, social, and financial resources but not objectively quantifiable material resources, with the purpose of encompassing a comprehensive and consistent array of resources that have been found to relate to lower psychological distress or more positive adjustment [11, 79]. Third, resources were assessed at T2 and T3 only and therefore, instead of the more immediate impact, our findings captured the delayed impact of COVID-19 on resources in its aftermath phase. Finally, our findings could be context-specific, with the high prevalence of resilience and small percentages on resource loss possibly related to the low incidence rate in Hong Kong throughout the pandemic. Future studies should investigate trajectories of psychiatric symptoms and their relationships with different patterns of changes in personal, social, and financial resources across countries and cultures, especially those more strongly affected by the pandemic in conjunction with other crises (e.g., social, financial, or military crises).

Notwithstanding the limitations, this study conducted statistically robust growth mixture modeling on a three-wave data collected from a population-representative sample, showing methodological and statistical rigor in investigating the prospective associations between resource changes and psychological outcome trajectories amid COVID-19. We studied three key types of resources (i.e., personal, social, financial), specifically their differential contributions to two most commonly studied psychiatric outcomes (i.e., depressive and anxiety symptoms). The current findings could also be applicable to improving public health interventions during global large-scale disasters and calling for attention from policymakers, scientists, and practitioners to establish or enrich the social ecology of the affected populations in order to maximize the odds of adaptive adjustment and reduce, in turn, additional burden on the already loaded healthcare system.

## Supplementary information

Supplementary Material
