# Supplementary material for: The interrelations between psychological outcome trajectories and resource changes amid large-scale disasters: A growth mixture modeling analysis
Source: Transl Psychiatry. 2023 Feb 15;13:57. doi: 10.1038/s41398-023-02350-4 (PMC9930711; doi:10.1038/s41398-023-02350-4)
Supplement: Supplementary file 1 — Supplementary Material [file 41398_2023_2350_MOESM1_ESM.docx]

**SUPPLEMENTARY MATERIAL 1** Sampling details.

**Detailed Sampling Procedure between February and July 2020 (T1)**

Upon obtaining the approval from the Ethics Committee of The Education University of Hong Kong, the Centre for Communication and Public Opinion Survey of The Chinese University of Hong Kong and Hong Kong Public Opinion Research institute were contracted to conduct telephone surveys at T1. Random digit dialing was employed based on a dual-frame sampling approach with both landline and mobile phone numbers (50% each) drawn from the databases released by the Hong Kong Communication Authority. Interviews were conducted with eligible respondents, who were (1) Hong Kong Chinese, (2) 15 years of age or older, and (3) Cantonese-speaking from 2 pm to 10 pm on both weekdays and weekends. Verbal informed consent was obtained prior to each interview. If multiple eligible members were identified in a successfully contacted household through landline phone calls, the one with the closest birthday to the interview date was selected. Further attempts were made for numbers with responses as “no answer”, “busy”, or “eligible respondent not at home”. A total of 8,063 respondents completed the surveys at T1 (February–March 2020, *n*=4,021; April–May 2020, *n*=2,008, July–August 2020, *n*=2,034).

**Detailed Sampling Procedure between March and August 2021 (T2)**

During March and August 2021 (i.e., T2 of the current study), a total of 1,964 T1 respondents were invited. 1,964 telephone numbers were attempted, 202 (10.29%) were ineligible for inclusion (i.e., invalid number or eligible respondents passed away) and the numbers of unknown eligible was 325 (16.55%). Among 1437 (73.17%) eligible successfully contacted numbers, 1,318 (91.72%) surveys were successfully completed online or through telephone interviews, and 119 (8.28%) refused.

**Detailed Sampling Procedure between September 2021 and February 2022 (T3)**

During September 2021 and February 2022 (i.e., T3 of the current study), a total of 1,333 T1 respondents were invited (including all 1,318 T2 respondents and 15 additional T2 non-respondents). 1,333 telephone numbers were attempted, 10 (0.75%) were ineligible for inclusion (i.e., invalid number) and the numbers of unknown eligible was 403 (30.23%). Among 920 (69.02%) eligible successfully contacted numbers, 906 (98.48%) surveys were successfully completed online or through telephone interviews, and 14 (1.52%) refused.

**Calculation Formulas**

| Response rate = | Completed / [Known eligibles + Unknown eligibles × Eligibles / (Eligibles + Ineligibles)] |
| --- | --- |
| Cooperation rate = | Completed / Known eligibles |

**SUPPLEMENTARY MATERIAL 2** Fit indices for growth mixture models for trajectories of depressive and anxiety symptoms.

| *Depressive symptoms* |  |  |  |  |  |
| --- | --- | --- | --- | --- | --- |
|  | 1 class | 2 classes | 3 classes | 4 classes | 5 classes |
| BIC | 20695.848 | 20443.544 | 20354.638 | 20289.907 | 20248.113 |
| SABIC | 20676.789 | 20414.955 | 20316.520 | 20242.259 | 20190.935 |
| Entropy | 1.000 | 0.787 | 0.797 | 0.805 | 0.733 |
| LMR-LRT (*p*-value) | –– | 261.763 (<.001) | 105.599 (.070) | 82.495 (.015) | 60.574 (.164) |
| BLRT (*p*-value) | –– | 273.889 (<.001) | 110.491 (<.001) | 86.317 (<.001) | 63.380 (<.001) |
| *Anxiety symptoms* |  |  |  |  |  |
|  | 1 class | 2 classes | 3 classes | 4 classes | 5 classes |
| BIC | 20573.392 | 20332.423 | 20195.423 | 20112.116 | 20053.522 |
| SABIC | 20554.333 | 20303.834 | 20157.307 | 20064.468 | 19996.344 |
| Entropy | 1.000 | 0.806 | 0.804 | 0.819 | 0.820 |
| LMR-LRT (*p*-value) | –– | 250.929 (<.001) | 151.562 (.002) | 100.251 (.002) | 76.630 (.127) |
| BLRT (*p*-value) | –– | 262.554 (<.001) | 158.583 (<.001) | 104.895 (<.001) | 80.180 (<.001) |

*Abbreviations*. BIC=Bayesian Information Criterion; SABIC=Sample-size Adjusted Bayesian Information Criterion; LMR-LRT=Lo-Mendell-Rubin Adjusted Likelihood Ratio Test; BLRT=Bootstrapped likelihood ratio test.
